# Molecular Mechanisms of Gonadotropin-Inhibitory Hormone (GnIH) Actions in Target Cells and Regulation of GnIH Expression

**DOI:** 10.3389/fendo.2019.00110

**Published:** 2019-02-25

**Authors:** You Lee Son, Takayoshi Ubuka, Kazuyoshi Tsutsui

**Affiliations:** ^1^Laboratory of Photobiology, Department of Ophthalmology, Keio University School of Medicine, Tokyo, Japan; ^2^Laboratory of Integrative Brain Sciences, Department of Biology and Center for Medical Life Science, Waseda University, Tokyo, Japan

**Keywords:** gonadotropin-inhibitory hormone/RFamide-related peptides (GnIH/RFRPs), GnIH receptor (GnIH-R), gonadotropin-releasing hormone (GnRH), kisspeptin, vasoactive intestinal polypeptide (VIP), gonadotropes, glucocorticoid (GC), thyroid hormone (TH)

## Abstract

Since gonadotropin-inhibitory hormone (GnIH) was discovered in 2000 as the first hypothalamic neuropeptide that actively inhibits gonadotropin release, researches conducted for the last 18 years have demonstrated that GnIH acts as a pronounced negative regulator of reproduction. Inhibitory effect of GnIH on reproduction is mainly accomplished at hypothalamic-pituitary levels; gonadotropin-releasing hormone (GnRH) neurons and gonadotropes are major targets of GnIH action based on the morphological interaction with GnIH neuronal fibers and the distribution of GnIH receptor. Here, we review molecular studies mainly focusing on the signal transduction pathway of GnIH in target cells, GnRH neurons, and gonadotropes. The use of well-defined cellular model systems allows the mechanistic study of signaling pathway occurring in target cells by demonstrating the direct cause-and-effect relationship. The insights gained through studying molecular mechanism of GnIH action contribute to deeper understanding of the mechanism of how GnIH communicates with other neuronal signaling systems to control our reproductive function. Reproductive axis closely interacts with other endocrine systems, thus GnIH expression levels would be changed by adrenal and thyroid status. We also briefly review molecular studies investigating the regulatory mechanisms of GnIH expression to understand the role of GnIH as a mediator between adrenal, thyroid and gonadal axes.

## Introduction

Gonadotropin-inhibitory hormone (GnIH) was initially isolated from the Japanese quail hypothalamus that inhibited gonadotropin release from the cultured quail anterior pituitary; this was the first demonstration of a hypothalamic neuropeptide directly inhibiting gonadotropin release in any vertebrate (1). GnIH peptides have since been identified in all vertebrate classes, and these share an LPXRFamide (*X* = L or Q) motif at their C-termini (2–4), thus also known as RFamide-related peptides (RFRPs). In mammals, GnIH precursor gene is translated and cleaved into at least two peptides, RFRP1 and 3 (2–4). Not only the presence of GnIH/RFRP peptides, but their function to inhibit gonadotropin secretion is also conserved across mammals, including mice, rat and humans (2, 3, 5–8).

Two G protein-coupled receptors, GPR147 and GPR74 have been identified as GnIH receptors (GnIH-Rs) (9–12). Yin et al. identified that membrane fraction of COS-7 cells transfected with quail GPR147 binds specifically to GnIH (12). Ikemoto and Park cloned GnIH-Rs in the chicken; GPR147 cDNA was only expressed in the brain and pituitary, whereas GPR74 cDNA was ubiquitously expressed in various tissues (11). In mammals, Hinuma et al. identified a specific receptor for RFRP and named it OT7T022, which was identical to GPR147 (10). Bonini et al. reported two GPCRs for neuropeptide FF (NPFF), which has PQRFamide motif at its C-terminal, NPFF1 (identical to GPR147) and NPFF2 (identical to GPR74) (9). From the higher GnIH binding affinity for GPR147 than GPR74, GPR147 is thought to be the principal receptor for GnIH (9, 11). GnIH-R couples to Gα_i_, which inhibits the activity of adenylate cyclase (AC), thus reducing intracellular cAMP levels and protein kinase A (PKA) activity (10, 13–15). Cell bodies of GnIH neurons are located in the paraventricular nucleus (PVN) in birds (1, 16, 17) and in the dorsomedial hypothalamic area (DMH) in most mammals (10, 18–21). The projection of GnIH neurons to gonadotropin-releasing hormone (GnRH) neurons is the most conserved property of GnIH neurons. GnIH neuronal axon terminals contact with GnRH neurons in axo-somatic as well as axo-dendritic contacts, that express GnIH-R in the preoptic area (POA) (18, 21–25). GnIH neuronal fibers are also observed in the median eminence to control anterior pituitary function via GnIH-R expressed in gonadotropes (1, 6, 7, 17, 22, 26, 27).

As reviewed elsewhere (2, 3, 8, 15, 28–31), much evidence now supports the notion of GnIH as a key neurohormone to inhibit reproduction by regulating the hypothalamic-pituitary function. Recent studies for deeper understanding of the detailed molecular mechanisms of GnIH action have reinforced the physiological significance of GnIH in reproductive regulation. Here, we address selective studies demonstrating the GnIH action mechanism uncovered by using cellular and molecular model systems.

## Potential Signaling Pathways That Convey the Inhibitory Action of GnIH in GnRH Neurons

### Regulators of GnRH Neuronal Function

GnRH is the final output of the brain that regulates reproduction by stimulating gonadotropin secretion, thus GnRH neuronal functions are finely tuned by various stimulatory and inhibitory signals. There is strong evidence supporting a direct suppressive effect of GnIH on GnRH neuronal activities. Direct application of GnIH to hypothalamic brain slices decreases the firing rate of a subpopulation of GnRH neurons (32) and a direct postsynaptic inhibition of GnRH neuronal firing may occur via GnIH-mediated hyperpolarization of K^+^ channels in vGluT2-GnRH neurons (33). Similarly, intracerebroventricular administration of GnIH suppresses c-Fos immunoreactivity in GnRH neurons (34).

Following the discovery of GnIH, kisspeptin, encoded by the *kiss1* gene (35), was demonstrated to play an important role in the up-regulation of the reproductive system in mammals (36–38). In contrast to GnIH actions, kisspeptin treatment potently activates electrical firing of GnRH neurons in hypothalamic slices (39, 40). Kisspeptin neurons make close contact with GnRH neurons acting at both the cell body and the nerve terminals (41, 42). The majority of GnRH neurons express the receptor for kisspeptin, GPR54 (43), which couples to Gα_q/11_ to activate phospholipase C and Ca^2+^ mobilization (44). Numerous studies have shown that kisspeptin acts as a key stimulatory regulator of the GnRH system (45).

Neurons synthesizing vasoactive intestinal polypeptide (VIP) are located in the suprachiasmatic nucleus (SCN) core sub-region and have monosynaptic connections with GnRH neurons (46, 47). GnRH neurons express the VIP/PACAP receptor subtype 2 (VPAC2) (48), which is preferentially coupled to the Gα_s_ signal transduction pathway that leads to accumulation of cAMP (49). VIP-targeted GnRH neurons preferentially express c-Fos during the afternoon of the luteinizing hormone (LH) surge on the day of proestrus (50, 51), and blocking VIP signaling *via in vivo* antisense antagonism abolishes GnRH/c-Fos activation in ovariectomized, estradiol-treated female rats (52, 53). Additionally, electrical responses of GnRH neurons to exogenous VIP exhibited peak activity around the predicted onset of the LH surge (54). Together, these lines of evidence suggest that VIP may facilitate GnRH release that leads to the preovulatory LH surge.

### Possible Interaction Between GnIH and Kisspeptin Signalings

**Figure 1:**
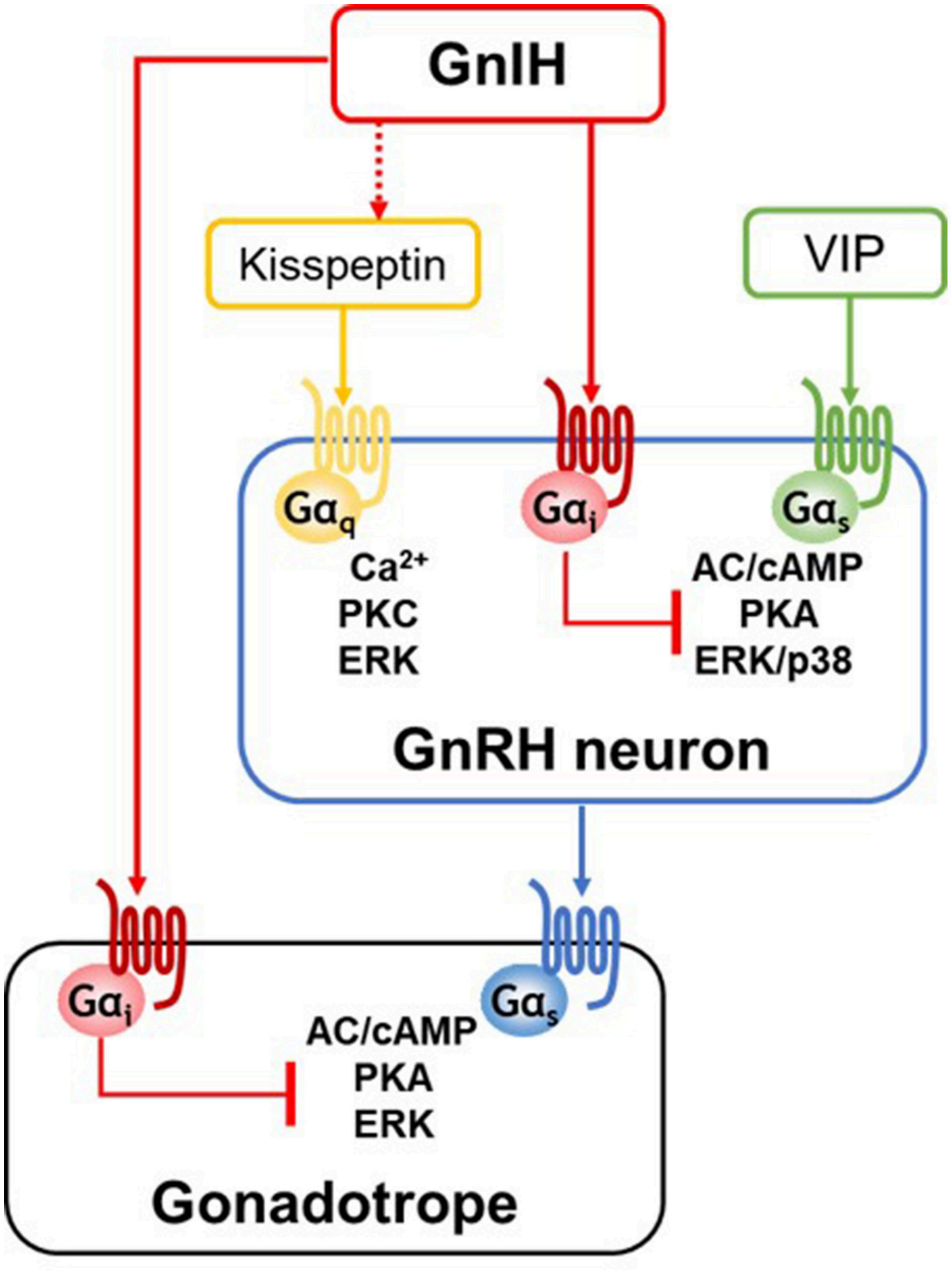
A schematic representation of GnIH action at the hypothalamic-pituitary levels. GnIH neurons project to hypothalamic GnRH neurons and pituitary gonadotropes, and GnIH directly acts via Gα_i_-coupled GPR147 or GPR74 expressed in its target cells. GnRH neurons are activated by kisspeptin or vasoactive intestine peptide (VIP) stimulation. GnIH has no direct inhibitory effect on kisspeptin/Gα_q_-coupled GPR54-induced Ca^2+^ or PKC pathway. Although kisspeptin/GPR54 pathway is not the direct target of GnIH action, GnIH may regulate kisspeptin neuronal activity via direct fiber contact and GnIH receptor expressed in kisspeptin neurons. On the other hands, GnIH effectively inhibits VIP/Gα_s_-coupled VPAC2-induced pathway by specifically acting on adenylate cyclase (AC)/cAMP/protein kinase A (PKA)-dependent pathway. In gonadotropes, GnIH exerts its inhibitory effect via AC/cAMP/PKA pathway, thus Gα_s_-coupled GnRH receptor signaling is specifically inhibited by GnIH.

From the opposite effects of kisspeptin and GnIH on the GnRH neuronal system, the interaction of their signal transduction pathways is expected to finetune the GnRH neuronal activity. The use of well-defined *in vitro* GnRH neuronal model system allows to examine their possible interaction occurring in GnRH neurons. GT1-7 is a clonal line of mature GnRH neurons of mouse hypothalamus (55). GT1-7 cells exhibit neuronal morphology with synapse formation and secrete mature GnRH in a pulsatile fashion, similar to GnRH neurons *in vivo* (55, 56). GT1-7 cells express GnIH-Rs, GPR147 and GPR74, as well as GPR54 (57–60), and the stimulatory effect of kisspeptin on GnRH system in GT1-7 cells has been demonstrated (57, 61–63). However, there was yet no evidence for direct inhibitory effect of GnIH on kisspeptin-induced signaling pathway in GnRH neurons. As the major downstream signaling events induced by kisspeptin/GPR54 in GT1-7 cells, Ca^2+^ mobilization-related nuclear factor of activated T-cells response element (NFAT-RE) activity and protein kinase C (PKC)-mediated extracellular-signal-regulated kinase (ERK)/mitogen activated protein kinase (MAPK) activity has been identified. However, it has been shown that GnIH has no inhibitory effect on these activities, even if GPR147 is overexpressed (60) (Figure 1). Although GnIH does not directly inhibit Gα_q/11_-mediated activities induced by kisspeptin in GT1-7 cells, there is strong evidence showing that GnIH may be involved in Ca^2+^ or PKC-related signaling pathway. Clarke et al. have found that ovine GnIH (RFRP3) potently blocks the generation of intracellular free Ca^2+^ in the pituitary elicited by GnRH, although they did not directly investigate the site of ovine GnIH action within the Ca^2+^ system (26). Reversely, Nichols *et al*. have shown that PKC inhibitor blocks human RFRP1 activity in cardiomyocytes, suggesting that RFRP1 activates PKC pathway to modulate cardiac contractile performance (64). Nevertheless, the effect of GnIH on kisspeptin-induced Ca^2+^ or PKC pathway has not yet been verified.

A study using another GnRH neuronal cell model, mHypoA-GnRH/GFP, generated from adult-derived GnRH-GFP neurons, shows the interaction of GnIH and kisspeptin on GnRH transcriptional regulation (65). In mHypoA-GnRH/GFP cells, treatment of GnIH attenuates basal GnRH mRNA expression, whereas kisspeptin induces GnRH levels. Co-treatment of GnIH and kisspeptin suppresses GnRH mRNA expression, suggesting the inhibitory effect of GnIH may override the stimulatory effect of kisspeptin on GnRH mRNA expression. By using transcriptional inhibitors (actinomycin D and DRB), Gojska et al. further show that GnIH-mediated repression is involved in new RNA synthesis rather than affecting the stability of pre-existing GnRH mRNA in mHypoA-GnRH/GFP cells (65). Although they present a novel action mechanism of GnIH on GnRH transcriptional regulation, these results do not indicate the direct inhibitory effect of GnIH on kisspeptin-induced signaling pathway.

Interestingly, it was shown that GnIH effectively suppresses kisspeptin-induced GnRH release in hypothalamic culture of adult mice (60). This phenomenon may be a result from the inhibition of GnIH effects on exocytosis of GnRH, not on kisspeptin/GPR54 signaling pathway in GnRH neurons. It would be also explained by the action of GnIH on kisspeptin neurons. Compared with GnRH neuronal cell models, there exist several neuronal networks for the actions of GnIH and kisspeptin controlling GnRH release in hypothalamic culture, similar to the *in vivo* environment. In this respect, GnIH may not directly interfere with the stimulatory effect of kisspeptin on GnRH neurons; but rather regulate the kisspeptin neuronal activity leading to GnRH release. It has been shown that ~25% of kisspeptin neurons in the arcuate nucleus express GPR147 or GPR74, where ~35% of arcuate kisspeptin cells received GnIH fiber contacts (66), suggesting a regulatory role of GnIH-mediated signaling in arcuate kisspeptin neurons.

### Inhibitory Action of GnIH on VIP/VPAC2 Signaling and its Physiological Significance

GT1-7 cells specifically express VPAC2 but not VPAC1 likewise GnRH neurons *in situ* and well respond to VIP stimulation (60, 61, 67, 68). Our recent study using GT1-7 cells clearly shows that GnIH suppresses the stimulatory effect of VIP at multiple levels, cAMP-response element (CRE) activity, ERK and p38 MAPK pathways, and *c-Fos* expression (60). In this study, the use of pharmacological inhibitor H89, which is specific to PKA pathway, but not the PKC inhibitor GF-109203X, results in an inhibition of VIP-induced pathways. Furthermore, it has been shown that ERK and p38 pathways activated by forskolin, which raise cAMP level, are effectively inhibited by GnIH, but GnIH has no inhibitory effect on PKC activator PMA (phorbol 12-myristate 13-acetate)-induced pathways (60), demonstrating the specific inhibitory action of GnIH on the cAMP/PKA pathway in GnRH neurons as in gonadotropes (Figure 1 and see also the section Specific inhibition of GnRH-induced signaling via cAMP pathway in gonadotropes by GnIH). Supporting this specific inhibitory role of GnIH on VIP-induced pathway shown in GT1-7 cells, GnIH eliminates the stimulated effect of VIP on GnRH release in female mouse hypothalamic explants (60).

It is hypothesized that VIP input is required for appropriate LH pulse frequencies and induction of an appropriately timed LH surge (52, 69, 70). The SCN of female rats, compared to males, have significantly greater VIP innervation of GnRH neurons (47), suggestive of a specific role for VIP in the regulation of estrous cycle. The necessity of VIP in triggering the afternoon GnRH surge has been also suggested (51, 52). These findings suggest that direct VIP projections from the SCN to GnRH system positively drive the GnRH/LH surge. Given the pronounced inhibitory actions of GnIH on VIP/VPAC2 signaling in GT1-7 cells and VIP-induced GnRH release (60), it seems probable that GnIH may regulate LH surge by suppressing VIP action on GnRH neurons. The inhibitory effect of GnIH on LH surge has been reported. Treatment of female rats with GnIH results in marked inhibition of GnRH neuronal activity at the time of LH surge (34) and intravenous infusion of GnIH blocks estrogen-induced LH surge in ewes (71). Henningsen et al. also showed that acute intracerebroventricular injection of GnIH just before the LH surge reduces the LH surge amplitude in female Syrian hamster (72). These findings have demonstrated the inhibitory role of GnIH on the amplitude of GnRH/LH surge, although the direct relationship between GnIH and VIP has not been investigated. Notably, SCN-derived VIP fibers project to GnIH neurons in female Syrian hamster, and central administration of VIP reduced c-Fos immunoreactivity in GnIH neurons in a time-dependent manner (73), indicating the possible SCN regulation of GnIH activity by VIP to control the timing of LH surge. Future studies are required to fully demonstrate the physiological relevance of interaction between GnIH and VIP on the timing and amplitude of GnRH/LH surge.

## Inhibitory Mechanism of GnIH Action in Gonadotropes

### Regulators of Pituitary Gonadotrope Activity

In addition to the role of GnIH at the hypothalamic level, GnIH neurons also project to the median eminence to control anterior pituitary function via GnIH-R expressed in gonadotropes (6, 7, 26, 27). On the other hand, there are relatively few or no GnIH fibers in some birds (74) and rodents (18, 21, 75), and GnIH has no direct inhibitory effect on LH secretion by the pituitary gonadotropes (34, 75, 76). Although there is some debate whether GnIH can directly act on the pituitary in some species, GnIH decreases the synthesis and/or release of pituitary gonadotropins, LH and follicle-stimulating hormone (FSH) in many species (24, 26, 77–80).

GnRH is the major activator of gonadotropes. Upon binding to its receptor (GnRH-R) on gonadotropes, GnRH stimulates the synthesis and release of LH and FSH (81). GnRH-R is a member of GPCR family (82). Most of the biological actions of GnRH are mediated by Gα_q/11_-coupled pathway (83). However, GnRH signaling may not be exclusively linked to Gα_q/11_-pathway, but also involve other pathways depending on the cell context (84). In fact, GnRH-R was shown to be coupled to Gα_s_ (85, 86). In primary pituitary culture, rat pituitary-derived G-GH3 cells and mouse gonadotrope LβT2 cells, GnRH-R couples to Gα_s_ as well as Gα_q/11_, whereas in αT3-1 pituitary precursor cells, CHO-K1 and COS-7 cells, GnRH-R seems to couple exclusively to Gα_q/11_ (87–89). Several studies have also suggested a physiological role of cAMP as a mediator of GnRH actions via Gα_s_-coupled pathway in the pituitary gland. A cell-permeable peptide that uncouples Gα_s_ from receptors is able to inhibit ERK and c-Fos activation, and LHβ expression in LβT2 cells, indicating that Gα_s_ is involved in GnRH-R signaling (86).

### Specific Inhibition of GnRH-Induced Signaling via cAMP Pathway in Gonadotropes by GnIH

It was shown that GnIH inhibits gonadotropin synthesis and/or release from cultured pituitaries in birds (1, 90) and mammals (79, 91, 92). Based on the characteristic of Gα_i_-coupled GnIH-Rs, it is expected that GnIH inhibits cAMP-related signaling pathways triggered by GnRH in gonadotropes. Using the LβT2 gonadotrope model system, the detailed mechanisms underlying the inhibitory effect of GnIH on gonadotropin synthesis has been investigated (93). LβT2 cells exhibit the characteristics of fully differentiated gonadotropes, including the expression of LH, FSH, and GnRH-R as well as displaying the appropriate responses to GnRH with dose-dependent increase in LH secretion (94–96). Furthermore, LβT2 cells express both GPR147 and GPR74 (59, 93), indicating that LβT2 is an appropriate cellular model system to investigate GnIH action occurring in gonadotropes.

In this study using LβT2 cells, GnRH treatment activates CRE activities, and GnIH effectively suppresses GnRH-induced CRE activities in a dose-dependent manner as well as cAMP production (93). GnIH also inhibits the downstream ERK phosphorylation via AC/PKA-dependent manner (Figure 1). The AC/cAMP/PKA-dependent inhibitory effect of GnIH are also demonstrated in GnRH-stimulated transcriptions of gonadotropin subunit genes, LHβ, FSHβ, and common α subunit. The inhibitory effect of GnIH on GnRH-induced CRE activity, ERK phosphorylation, and gonadotropin expression leads to reduction in LH levels in LβT2 cells. This study suggests that as in GnRH neurons (described in section Inhibitory action of GnIH on VIP/VPAC2 signaling and its physiological significance), GnIH specifically acts via cAMP pathway in its target cells (93).

## Common GnIH Inhibitory Mechanism in its Target Cells

From the identification of GPR147 and GPR74 as GnIH-Rs (9–12), suppression of cAMP production by GnIH has been shown in several studies (10, 13, 14). The precise mechanism of GnIH action in hypothalamic GnRH neurons as well as pituitary gonadotropes has been investigated in the cellular model systems through the molecular approaches on GPCR-related second messenger activity, downstream MAPK cascade, and the effect of pharmacological modulators. The results obtained by analysis of signaling pathway suggest that GnIH may play as a brake by preventing the excessive action of stimulatory inputs to maintain the balance in reproductive system. As a conserved mechanism of GnIH action, the AC/cAMP/PKA-specific inhibitory pathway has been demonstrated in hypothalamus-pituitary levels (60, 93) (Figure 1). Therefore, GnIH may govern the hypothalamic neuronal activities of GnRH by inhibiting the action of VIP and kisspeptin directly or indirectly, thus eventually reduce pituitary gonadotropin secretion. However, there are several exceptions of the GnIH effect on GnRH/LH release. There is a report showing that acute central injection of GnIH induces c-Fos expression in GnRH neurons and increases LH, FSH, and testosterone secretion in Syrian hamster (76). Similarly, GnIH has shown the dose-dependent stimulatory effect on LH secretion in adult male mice (97). In male Siberian hamsters, central administration of GnIH inhibits LH release in long day photoperiods, whereas stimulates LH release in short day, indicating that GnIH peptides finely tune LH levels in an opposite fashion across the seasons (21). Based on the complex regulation of GnIH action depending on the species/sexes, seasons and reproductive stages, future research is needed to determine when and how GnIH exerts its inhibitory or stimulatory effect in target cells.

## Regulatory Mechanism of GnIH Expression by Glucocorticoid and Thyroid Hormone

Considering the role of GnIH as an upstream regulator of the hypothalamic-pituitary-gonadal (HPG) axis, abnormal GnIH expression levels may cause reproductive dysfunctions. Therefore, we discuss some endocrine regulators leading to GnIH expressional changes. See the recent review (31) for the regulatory mechanism of GnIH expression by melatonin and photoperiod (19, 21, 98).

### Molecular Mechanism of Glucocorticoid-Mediated GnIH Activation

Reproductive function is suppressed under stress (99–101), suggesting the interaction between hypothalamic-pituitary-adrenal (HPA) and HPG axes. From the inhibitory role of GnIH in reproduction, the GnIH system could be a good candidate mediating stress-induced reproductive dysfunction. Supporting this, there have been several studies showing that stress activates the GnIH system in birds and mammals. In adult house sparrows, capture-handling stress shows a significant increase in the number of GnIH neurons (102). In rat, immobilization stress leads to an up-regulation of GnIH expression (103) and stressful stimuli increase the expression of c-Fos protein in GnIH neurons of the DMH (104). These results suggest that suppressive effects of stress upon reproductive functions are mediated by the hypothalamic GnIH system. The inhibitory effect of stress on reproductive function is potentially mediated by high concentrations of circulating glucocorticoids (GC) acting via the GC receptor (GR) (105, 106). In adult rat, approximately half of hypothalamic GnIH neurons express GR (103). In quail, most GnIH-positive cells express GR mRNA and 24 h treatment with corticosterone (CORT) increases GnIH mRNA expression (107).

**Figure 2:**
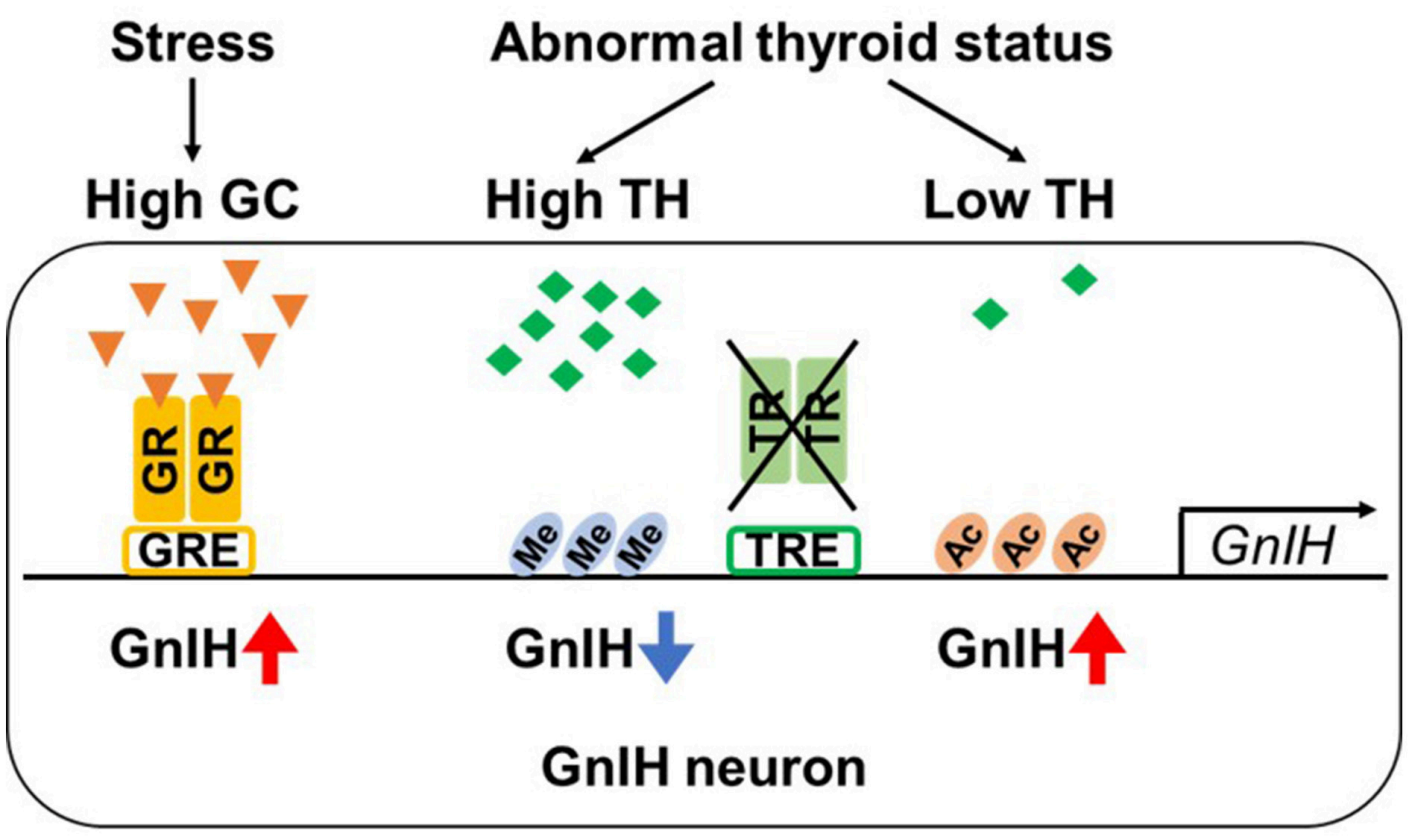
Regulation of GnIH promoter activity by glucocorticoid and thyroid hormone. GnIH expression is regulated by glucocorticoid (GC). GnIH neurons express GC receptor (GR) and GC-response element (GRE) is present in GnIH promoter region. Stress increases GC levels, and GC acts by binding to GR. When GC-bound GR is recruited to GRE, GnIH expression is up-regulated. GnIH expression is also actively changed by concentration of thyroid hormone (TH). Although GnIH neurons express TH receptors (TRα and β) and putative TH-response elements (TREs) exist in GnIH promoter region, TRs do not directly bind to GnIH promoter. However, thyroid status highly regulates the chromatin modification of GnIH promoter. Hypothyroidism exhibits increased GnIH expression with hyperacetylation of H3 (Ac) in promoter region. On the other hand, hyperthyroidism decreases GnIH expression associated with H3K9tri-methylation (Me).

Using a GnIH-expressing neuronal cell line, rHypoE-23 derived from rat hypothalamus (108), the detailed molecular mechanism of GC-mediated GnIH transcriptional activation has been investigated (107). rHypoE-23 cells express GR mRNA and GnIH mRNA expression is activated by 24 h CORT treatment. There exist several glucocorticoid response elements (GREs) in the upstream of rat GnIH precursor coding region. Through promoter analysis, it has been identified that −1,530 bp GRE is critical for the CORT-stimulated GR recruitment and its transcriptional activity (107) (Figure 2). This study provides a putative molecular basis for transcriptional activation of GnIH under stress by demonstrating that CORT directly induces GnIH transcription by recruitment of GR to its promoter. Another study using the rHypoE-23 GnIH neuronal cells has shown that the GC agonist, dexamethasone (DEX), which directly acts on GR, increases GnIH and GPR147 mRNA levels (109). The effect of neonatal DEX exposure on reproductive maturation has been also investigated in female mice (110). DEX-treated females have exhibited delayed pubertal onset and irregular estrus cycles with decreased GnRH mRNA expression in the POA and increased GnIH cell numbers in the DMH, suggesting that DEX-mediated activation of GnIH system may lead to inhibition of GnRH expression.

### Thyroid Hormone-Mediated GnIH Regulation by Chromatin Modification

Recently, thyroid hormones (THs; thyroxine, T_4_ and triiodothyronine, T_3_) have been suggested as a novel hormonal regulator of GnIH expression (111, 112). THs play an important role in proper development and function of the reproductive system, particularly in pubertal onset (113, 114), indicating interactions between the hypothalamic-pituitary-thyroid (HPT) and HPG axes. Therefore, thyroid disorders such as hypothyroidism and hyperthyroidism, cause abnormal puberty (115–117). Kiyohara et al. showed that hypothyroidism induced by long-term administration of propylthiouracil (PTU) in juvenile female mice leads to delayed pubertal onset with increased GnIH expression and reduced pituitary-gonadal activity, and knockout of GnIH prevents the effect of hypothyroidism to delay the pubertal onset. In contrast, hyperthyroidism induced by T_4_ leads to a decrease in GnIH expression, although pubertal onset was normal. Further, T_3_ treatment suppresses GnIH mRNA expression in hypothalamic explants. Although GnIH neurons express TH receptors, TRα and TRβ, and putative TH-response elements (TREs) are present in mouse GnIH promoter, TRs do not directly bind to GnIH promoter (111). As the molecular mechanism by which different TH concentration results in GnIH expressional changes, Kiyohara et al. have also demonstrated that the thyroid status highly regulates the chromatin modifications of GnIH promoter to activate and repress GnIH expression by H3acetylation and H3K9tri-methylation, respectively (Figure 2). Although to date limited information is available for the TH-mediated GnIH regulation, this study indicates a novel function of GnIH to mediate HPT-HPG interactions that contribute to proper pubertal development.

## Conclusion

The endocrine systems, HPA, HPG and HPT axes are closely connected, thus hormonal imbalance leads to reproductive dysfunctions. As a key hypothalamic inhibitor, GnIH may act on the most upstream level of the HPG axis by regulating the hypothalamic GnRH and kisspeptin neurons as well as pituitary gonadotrope activity. The significance of GnIH system on reproduction has been emphasized by identifying the novel function of GnIH system and its interaction with other endocrine systems of HPA and HPT via GC and TH, respectively. Changes in GnIH expression levels by these endocrine modulators will alter GnRH neuronal activity and gonadotropin release by specifically acting on AC/cAMP/PKA pathway. Based on the complex regulatory system of endocrine interactions, it is also expected to uncover a novel involvement of GnIH system in reproductive regulation. The precise molecular mechanism for GnIH action and identification of molecular target for GnIH regulation may contribute to the development of new pharmaceuticals.

## Author Contributions

YLS wrote the manuscript. TU and KT edited the manuscript.

### Conflict of Interest Statement

The authors declare that the research was conducted in the absence of any commercial or financial relationships that could be construed as a potential conflict of interest.
